# Neurological Complications of Coronavirus Disease (COVID-19): Encephalopathy, MRI Brain and Cerebrospinal Fluid Findings: Case 2

**DOI:** 10.7759/cureus.7930

**Published:** 2020-05-02

**Authors:** Patricio S Espinosa, Zufe Rizvi, Pamraj Sharma, Fawzi Hindi, Asia Filatov

**Affiliations:** 1 Neurology, Marcus Neuroscience Institute, Boca Raton Regional Hospital, Boca Raton, USA; 2 Neurology, Charles E. Schmidt College of Medicine, Florida Atlantic University, Boca Raton, USA

**Keywords:** covid 19

## Abstract

The neurological complications of coronavirus disease 2019 (COVID-19) are being better understood as the pandemic progresses. We report a second case of a patient who presented with COVID-19 infection and encephalopathy to our institution. In addition, we report MRI brain and cerebrospinal fluid data. COVID-19 does not seem to cross the blood-brain barrier. The exact mechanisms of encephalopathy and pathological response of COVID-19 are unknown.

## Introduction

Coronavirus disease (COVID-19) is a pandemic with more than 1,600,000 reported cases worldwide to date [1,2]. The neurological complications of COVID-19 are reported as the epidemic unfolds. Neurological ailments such as confusion and altered mental status have been circulating in case reports. However, evidence regarding neurological manifestation is scarce. By now, the population is familiar with the typical hallmarks of COVID-19: fever, cough and shortness of breath. However, stranger symptoms have been making the frontlines. Our institution has muddled through a case of COVID-19 encephalopathy, where a plethora of neuroimaging and spinal tap came back normal without signs of infection and other causes such as delirium were ruled out. We present a second patient with encephalopathy and COVID infection that presented to our institution, concluding that severe acute respiratory syndrome coronavirus 2 (SARS-COV-2) does not cross the blood-brain barrier. This article adds important brain imaging and cerebrospinal fluid (CSF) data about the neurological complications of this pandemic.

## Case presentation

**Figure 1:**
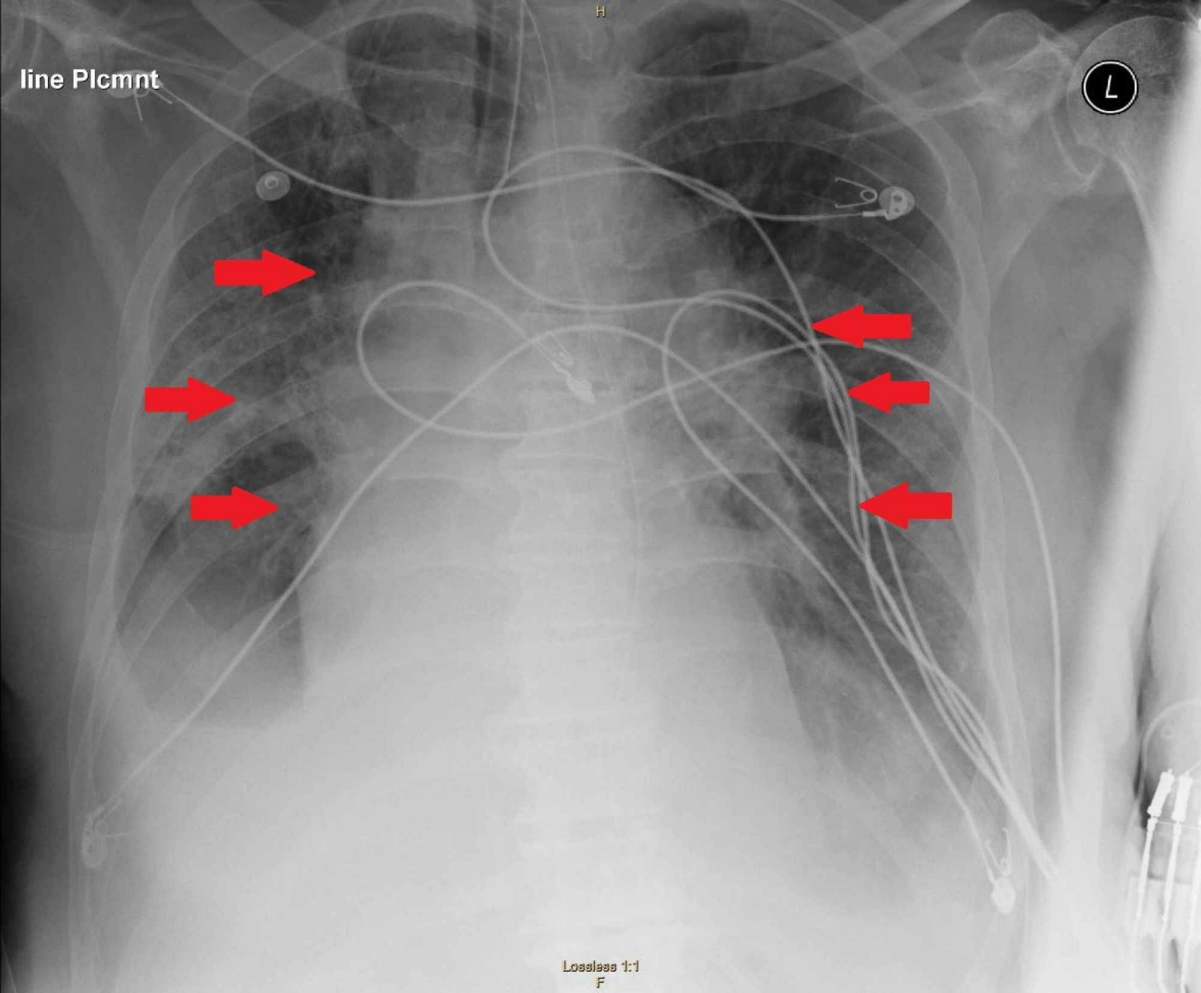
Chest x-ray demonstrating multifocal consolidations concerning for multifocal pneumonia.

A 72-year-old male with a past medical history of hypertension, hyperlipidemia and type 2 diabetes mellitus presented to the emergency department with a chief complaint of fever and dry cough. The patient reported the symptoms occurred after he came in contact with his neighbor who was a confirmed COVID-19 case, six days prior to admission. All protective measures and precautions for suspected COVID-19 infection were taken. The patient was placed in droplet precautions and contact isolation. Both sputum and nasopharyngeal cultures were negative for Strep. Blood cultures were negative, and urine analysis was negative. Influenza A and B tests were negative. His chest x-ray revealed multifocal consolidations concerning for multifocal pneumonia (see Figure 1).

**Figure 2:**
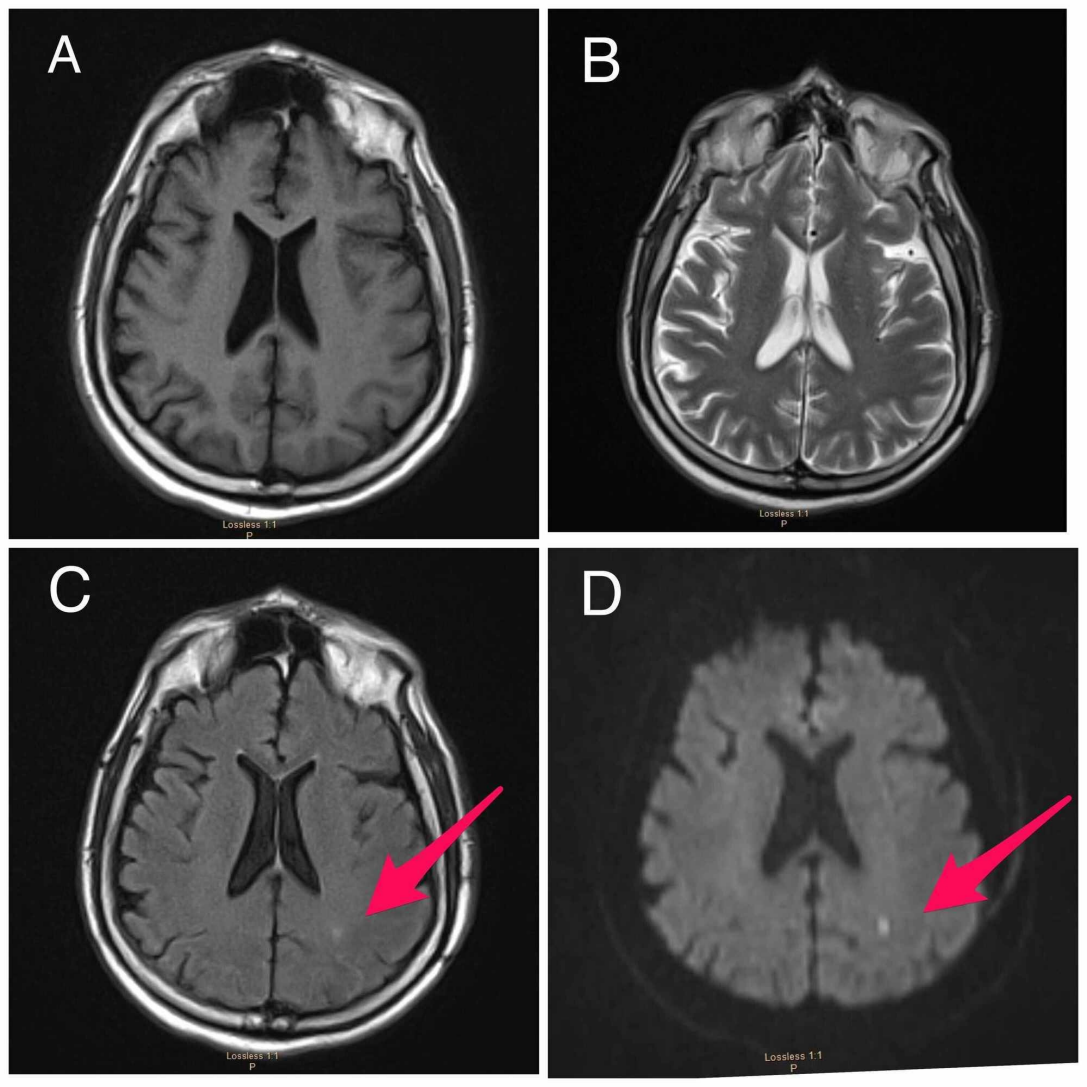
MRI of the brain. (A) Normal T1-weighted, (B) normal T2-weighted, (C) FLAIR sequence shows increased signal in the area of the acute stroke (arrow) and (D) diffusion-weighted imaging shows an area of restricted diffusion in the left parietocoritcal region (arrow). FLAIR, fluid attenuated inversion recovery.

The patient continued to decompensate and was ultimately intubated for acute hypoxemic respiratory failure. Hospital course was complicated by shock, requiring vasopressors. MRI brain (see Figure 2) revealed a small incidental finding of a cortical posterior left parietal infarct due to hypoperfusion in the setting of hypotension.

**Figure 3:**
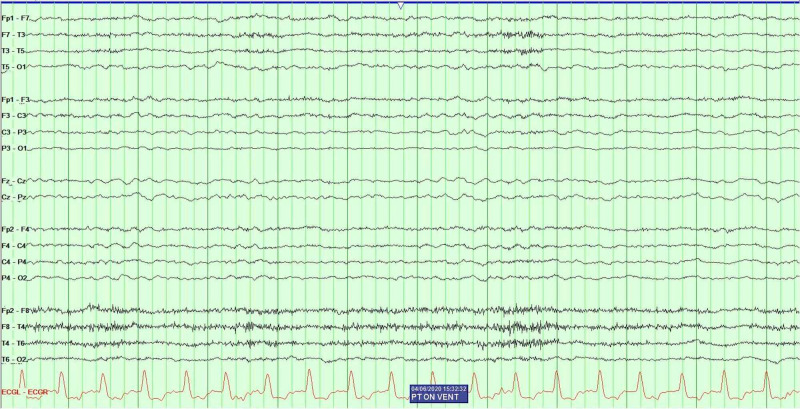
Electroencephalography revealed diffuse slowing and no evidence of epileptiform abnormalities.

SARS-CoV-2 polymerase chain reaction (PCR) testing resulted positive. Routine labs recommended by CDC for COVID-19 were obtained. Serum alanine aminotransferase level 730 U/L (normal range 10-130 U/L), aspartate aminotransferase level 735 U/L (normal range 10-34 U/L), lactate dehydrogenase 956 U/L (normal range 140-280 U/L), C-reactive potein 10.20 mg/L (normal range 0-10 mg/L), ferritin 6,137 ng/mL (normal range 12-300 ng/mL), D-dimer 12.73 (mg/L) (a normal D-dimer is considered less than 0.50 mg/L) and IL-6 20 pg/mL (normal range 5-15 pg/mL). The patient completed a course of ceftriaxone, azithromycin and five days with hydroxychloroquine. After being on the ventilator for 10 days, the patient oxygenation requirements gradually improved and his lung mechanics were almost normal; hence, ‘sedation awakening trials' were initiated for spontaneous breathing trials, but he was persistently not arousable. The patient remained hemodynamically unstable and ventilator dependent; however, sedation was discontinued while the patient did not show any improvement neurologically. The patient did not respond to verbal command or react to noxious painful stimuli. Furthermore, only his brainstem reflexes remained intact by grimace. In order to investigate the diagnosis of encephalopathy, further diagnostics were warranted. An electroencephalogram (EEG) (see Figure 3) was performed after the patient was off sedation for 72 hours and showed only bilateral slowing consistent with encephalopathy and no evidence epileptiform activity.

Lumbar puncture was performed with CSF analysis (see Table 1). CSF analysis showed no evidence to suggest meningitis or encephalitis. Case 1, a previously published case of COVID-19 with encephalopathy, is compared with our current case which is similar in CSF findings [1]. 

**Table 1:** CSF analysis: CSF studies show no evidence of CNS infection. CMV, cytomegalovirus; CNS, central nervous system; CSF, cerebrospinal fluid; HSV, herpes simplex virus; PCR, polymerase chain reaction; RBC, red blood cell; RSV, respiratory syncytial virus; WBC, white blood cell.

CSF studies	Case 1	Case 2
Appearance	Clear	Clear
Color	None	None
WBCs	4	2
RBCs	0	0
CSF glucose	75	87
CSF protein	68	27
HSV PCR	Not detected	Not detected
CMV PCR	Negative	Negative
RSV	Negative	Negative

## Discussion

Patients with COVID-19 develop encephalopathy; the virus does not seem to cross the blood-brain barrier, yet this is the second case of encephalopathy associated with the SARS-CoV-2 virus in our center. CSF analysis is within normal limits. MRI of our patient with encephalopathy and COVID-19 shows no evidence consistent with structural damage to the brain. The small border zone stroke found in brain imaging is likely secondary to hypotension in this critically ill patient and is not related to the virus. MRI is a power tool in a patient with COVID-19; in this case, it assisted in the decision of continuing care versus withdrawing care. EEG is a very helpful tool in patients with encephalopathy. CSF analysis is crucial in a patient with this infection as it helps rule other causes of altered mental status.

These cases add to the body of literature that patients with this virus have encephalopathy and no signs of meningitis, encephalitis, acute inflammatory demyelinating polyradiculopathy, myositis, critical illness myopathy, Guillain-Barre syndrome or anosmia [3,4]. The cause of encephalopathy is likely multifactorial, yet the virus may contribute to the encephalopathy. Anecdotally it has been reported that the encephalopathy may be secondary to neurotropism through the olfactory bulb and/or hematogenous spread [5]. However, these theories have yet to be proven since CSF analysis of virus PCR cannot be done at this time, despite several attempts to contact multiple labs and institutions across the USA. In patients with COVID-19 infection and encephalopathy, medical staff should consider the possibility of nervous system infections and carry out CSF tests in time to avoid delayed diagnosis and further reduce the mortality rate of critically ill patients.

## Conclusions

In our second patient with COVID-19 infection, there is no evidence of central nervous system penetration of the virus. We hypothesized that the likely cause of the encephalopathy in these patients is multifactorial. The aim of this abstract is to add to the literature information about patients with neurological complications and COVID-19, using real-time data. It is necessary to ensure that all clinicians are treating patients with the best data for the best patient outcome. As the body of literature grows, this article and others like this will help identify patterns of neurological illness amongst COVID-19 patients. 
